# Uric acid is associated with adiposity factors, especially with fat mass reduction during weight loss in obese children and adolescents

**DOI:** 10.1186/s12986-020-00500-9

**Published:** 2020-09-22

**Authors:** Yang Niu, Xue-lin Zhao, Hui-juan Ruan, Xiao-meng Mao, Qing-ya Tang

**Affiliations:** 1 Department of Clinical Nutrition, Xinhua Hospital, School of Medicine, Shanghai Jiao Tong University, Shanghai, 200092 China; 2 Shanghai Key Laboratory of Pediatric Gastroenterology and Nutrition, Shanghai, 200092 China

**Keywords:** Children, Obese, Uric acid, Body composition, Fat mass

## Abstract

**Background:**

Current adult studies suggest that uric acid (UA) is associated with body fat, but the relationship in obese children is unclear. Thus, we aim to evaluate the association between uric acid and body composition of obese children.

**Methods:**

A total of 79 obese children were included in this study, and 52 children (34 boys and 18 girls) underwent a 6-week weight loss camp, including 34 boys and 18 girls. Six-week weight-loss interventions were performed on all participants through aerobic exercise and appropriate dietary control. Laboratory tests and body composition were collected before and after the intervention.

**Results:**

Before the intervention, correlation analysis demonstrated that uric acid was positively correlated with height, weight, body mass index (BMI), waist circumference, hip circumference, fat mass (FM), and free fat mass (FFM) with adjusting for age and gender (*P* < 0.05). After 6 weeks of intervention, the participants gained 3.12 ± 0.85 cm in height, body fat percentage decreased by 7.23 ± 1.97%, and lost 10.30 ± 2.83 kg in weight. Univariate and multivariate analysis indicated that uric acid at baseline was associated with FM reduction during weight loss (*P* < 0.05).

**Conclusions:**

This study is the first report that uric acid is associated with BMI and FM, and may play an important role in the reduction of FM during weight loss in obese children and adolescents. The interaction between UA and adiposity factors and its underlying mechanisms need to be further explored.

**Trial registration:**

This study was registered in Clinical Trials.gov (NCT03490448) and approved by the Ethics Committee of Xinhua Hospital, Shanghai Jiao Tong University School of Medicine.

## Background

In 2014, a large-scale study of the world's obese population revealed that the number and proportion of obese and overweight people worldwide has been increasing in the past 30 years [1]^.^ The total global obese and overweight population has increased from 857 million in 1980 to 2.1 billion in 2013, and the number of obese and overweight children has increased by 47.1% [1]. Obesity has been defined as a worldwide epidemic metabolic disease by the World Health Organization (WHO), and it has become a worldwide public health problem endangering human health [2]. Childhood obesity not only leads to an increase in the incidence of chronic diseases such as fatty liver, diabetes, lipid metabolism disorders, and hypertension, but also easily leads to psychological problems such as inferiority and social disorders.

Hyperuricemia, previously thought to occur in adults, is also increasing in children, especially in obese children. The United States of American Bogalusa Heart Research Institute found that the incidence of hyperuricemia in boys and girls of normal weight was 8.1% and 8.5%, respectively, while that of obese children increased to 24.6% and 23.9% [3]. In present, a large number of current studies are exploring the relationship between serum uric acid and body mass index (BMI), glucose and lipid metabolism and blood pressure in children [4–7]. However, BMI has some limitations because body composition is not taken into account. Studies in adults have shown that blood uric acid levels are related to body composition and may be affected by fat mass (FM) and free fat mass (FFM), which are the major components of body weight [8, 9]. But no studies have explored the associations between FFM, FM, and serum uric acid in obese children and adolescents.

Therefore, this study is to explore the potential relationship between blood uric acid levels and FFM and FM, and the effect of serum uric acid on FFM and FM during weight loss in obese children and adolescents.

## Materials and methods

### Participants

A total of 79 obese children and adolescents who participated in the 6-week weight loss camp in Shanghai from July to August 2014 were selected as the research subjects. All participants and their parents signed written informed consent. All enrolled children had no prior liver and kidney damage and had no history of using drugs that affected uric acid. According to the WHO standards, obesity is diagnosed when it equals or exceeds the 95th percentile (P95) of the BMI of children of the same age and gender [10].

### Interventions and methods

A 6-week weight loss program was conducted for all participants under the guidance of professional weight loss coaches and medical staff, and weight loss was performed according to a unified exercise and diet program. Prior to the intervention, all subjects were required to undergo an exercise stress test to ensure safe and effective physical exercise. ① Sports programs: Sports programs mainly included ball sports such as basketball, table tennis, badminton, as well as aerobic sports such as jogging, brisk walking, power cycling, and swimming. All sports were carried out indoors under the guidance of a full-time coach. Exercises are performed 6 days a week, once in the morning and afternoon, and 2 h each time. Preparatory activities were performed for 15–20 min before and after the exercise. During exercise, the participants' heart rate was monitored though the sports wrist watch to ensure that the participant performs a small-medial load aerobic exercise. ② Diet plan: Considering the needs of children's growth and development, the diet should guarantee the daily energy and physiological requirements, and the basal metabolic rate (BMR) is calculated according to the Harris–Benedict formula to formulate the diet. The diet consists of 20% protein, 30% fat and 50% carbohydrates. In the menu provided to the participants, we increased the amount of coarse grains and vegetables and reduced the intake of high-fat foods. All foods were mainly cooked by steaming, boiling, and cold and dressed with sauce, rather than fried. All subjects did not take any type of nutritional supplement during weight loss.

### Physical measurements and laboratory examination

Before and after the intervention, all participants were measured for height (Seca264, Germany), weight (Inbody 370, Korea), waist circumference (WC), hip circumference (HC) ( Measuring with a tape measure), and systemic blood pressure (SBP) and diastolic blood pressure (DBP) (HEM-7052, Omron, Japan) in the morning and fasting state. BMI, waist-to-hip ratio (WHR) and waist-to-height ratio (WHtR) were calculated as follows: BMI (kg/m^2^) = (weight in kg)/(height in meters)^2^; WHR = waist circumference (cm)/hip circumference (cm); WHtR = waist circumference (cm)/height (cm). We evaluated participant's body fat percentage (BFP), FM, and FFM using bioelectrical impedance analyses (BIA) by whole-body impedance (Inbody 370, Korea) in the morning and fasting state.

In the same state, participants' serum uric acid (UA), fasting blood glucose (FBG), fasting insulin (FINS), triglyceride (TG), total cholesterol (TC), high density lipoprotein-cholesterol (HDL-C), and low density lipoprotein-cholesterol (LDL-C) were measured and collected. All laboratory indicators were tested by Aidikang Medical Test Center, FBG is tested by HK method, FINS was detected by chemiluminescence method, HDL-C and LDL-C were detected by homogeneous assay method, TC was detected by cholesterol oxidase method, TG Enzymatic GPO-POD was used for detection, and UA was analyzed using urase method. Homeostasis model assessment‐insulin resistance (HOMA‐IR) and Homeostasis model assessment‐β (HOMA‐β) were calculated as follows: HOMA‐IR = FBG (mmol/L) × FINS (mIU/L)/22.5; HOMA‐β = 20 × FINS (mIU/L)/[FBG (mmol/L)‐3.5]%. The relative changes (Δ) = the value after intervention − the value before intervention. Therefore, in the expression of the results, a positive value indicates a increase, and a negative value indicated an decrease.

### Statistical analysis

SPSS V.25.0 statistical software was used for statistical processing. Normal distributions of parameters were assessed using the Kolmogorov–Smirnov test. For the continuous variables, the independent sample t test was used, which was expressed as the mean ± standard deviation. For the categorical variables, the chi-square test and percentage (%) were used. Non-normally distributed data were presented as median (P25, P75). Firstly, univariate analysis was used to analyze the correlation between uric acid before intervention and relative changes of uric acid and variables. Considering that children's uric acid was related to age and gender [11–13], age and gender were adjusted while analyzing the correlation between uric acid and various indicators.

Secondly, in order to further analyze the correlation between the reduction of body fat and various indicators at baseline, participants were divided into two groups according to fat mass reduction during weight loss: an ΔFM < 10 kg group (n = 29), and an ΔFM ≥ 10 kg group (*n* = 23). The correlation between FM and other factors was assessed using the Pearson correlation, partial correlation analysis was used with adjusting for age and gender. Multiple linear regression analysis was conducted to further evaluate the impacts of the associated variables. A two-sided *P* value < 0.05 was considered statistically significant.

## Results

### General data and factors associated with uric acid before and after the intervention

Initially, a total of 79 obese children (52 boys and 27 girls) with the mean age of 13.13 ± 2.15 years (9–18 years old) participated in the weight loss camp. Before the intervention, correlation analysis demonstrated that serum uric acid was positively correlated with height, weight, BMI, WC, HC, FM, FFM, TC, and TG (All *P* < 0.05, Table 1). Furthermore, after adjusting for age and gender, there were still significant correlations between UA and BMI, HC, FM, FFM, TC, and TG (*P* < 0.05, Table 1).

**Table 1:** The correlation between uric acid at baseline and variables before intervention

	Before intervention (n = 79)	Uric acid (mmol/L)		Adjustment for age and sex	
		*r*	*P*	*r*	*P*
Height (cm)	163.89 ± 10.09	0.430	< 0.001	0.344	0.002
Weight (kg)	85.28 ± 20.77	0.447	< 0.001	0.381	0.001
BMI (kg/m^2^)	31.31 ± 4.78	0.360	0.001	0.281	0.013
WC(cm)	98.85 ± 14.77	0.311	0.005	0.162	0.159
HC (cm)	105.35 ± 10.58	0.363	0.001	0.334	0.003
WHR	0.94 ± 0.11	0.083	0.469	− 0.056	0.628
WHtR	0.60 ± 0.07	0.154	0.174	0.045	0.695
FBG (mmol/L)	4.91(4.69, 5.15)	0.131	0.250	0.102	0.379
FINS (pmol/L)	132.33 ± 58.27	− 0.085	0.456	− 0.092	0.426
HOMA-IR	4.14 ± 1.75	− 0.081	0.479	− 0.091	0.431
HOMA-β (%)	532.84 ± 246.25	− 0.056	0.622	− 0.073	0.526
TC (mmol/L)	4.49 ± 0.89	0.226	0.045	0.255	0.025
TG (mmol/L)	1.61 ± 0.80	0.358	0.001	0.337	0.003
HDL (mmo/L)	1.18 ± 0.26	− 0.204	0.071	− 0.166	0.150
LDL (mmol/L)	2.88 ± 0.68	0.227	0.044	0.221	0.053
UA (mmol/L)	453.42 ± 104.44	–	–	–	–
FM (kg)	36.13 ± 10.84	0.336	0.003	0.257	0.024
FFM (kg)	49.13 ± 11.87	0.476	< 0.001	0.450	< 0.001
BFP (%)	42.04 ± 5.46	− 0.020	0.863	0.021	0.856
SBP (mmHg)	120.33 ± 12.01	0.372	0.001	0.290	0.011
DBP (mmHg)	69.65 ± 9.25	0.245	0.030	0.180	0.117

However, Only 52 children (34 boys and 18 girls) underwent a 6-week weight loss camp, with the mean age of 13.21 ± 2.20 years (9–17 years old). After 6 weeks of intervention, the indicators after the intervention were better than those before the intervention. After adjusting for age and gender, UA was associated with BMI, HC, and FFM (*P* < 0.05, Table 2), but not FM (*P* > 0.05, Table 2).

**Table 2:** The correlation between uric acid and variables after intervention

	After intervention (n = 52)	Uric acid (mmol/L)		Adjustment for Age and Sex	
		*r*	*P*	*r*	*P*
Height (cm)	167.56 ± 8.79	0.432	0.001	0.194	0.176
Weight (kg)	76.82 ± 17.22	0.471	< 0.001	0.317	0.025
BMI (kg/m^2^)	27.07 ± 3.99	0.396	0.004	0.298	0.035
WC(cm)	88.90 ± 10.76	0.441	0.001	0.296	0.037
HC (cm)	97.83 ± 8.40	0.407	0.003	0.344	0.015
WHR	0.91 ± 0.07	0.238	0.089	0.112	0.440
WHtR	0.53 ± 0.05	0.300	0.031	0.255	0.074
FBG (mmol/L)	4.54 ± 0.35	0.042	0.768	0.001	0.995
FINS (pmol/L)	52.87 ± 20.74	0.128	0.367	0.134	0.354
HOMA-IR	1.54 ± 0.62	0.130	0.360	0.134	0.352
HOMA-β (%)	124.19 ± 59.96	0.110	0.437	0.103	0.478
TC (mmol/L)	3.51 ± 0.45	− 0.066	0.640	0.115	0.427
TG (mmol/L)	0.75 ± 0.20	0.146	0.301	0.191	0.185
HDL (mmo/L)	1.06 ± 0.19	− 0.322	0.020	− 0.236	0.099
LDL (mmol/L)	2.35 ± 0.40	0.055	0.701	0.236	0.098
UA (mmol/L)	419.25 ± 87.83	–	–	–	–
FM (kg)	27.59 ± 9.40	0.266	0.057	0.196	0.172
FFM (kg)	49.22 ± 10.56	0.532	< 0.001	0.361	0.010
BFP (%)	35.52 ± 6.44	− 0.093	0.513	0.045	0.755
SBP (mmHg)	114.27 ± 10.63	0.274	0.050	0.165	0.252
DBP (mmHg)	61.98 ± 9.02	0.131	0.356	0.089	0.541

As for the changes before and after intervention, the participants gained 3.12 cm in height, body fat percentage decreased by 7.23%, and lost 10.30 kg in weight (Table 3). Weight loss is dominated by a decrease in fat mass (9.92 kg), but with little increase in free fat mass (0.28 kg) (Table 3). The correlation analysis indicated that serum uric acid before intervention was negatively correlated with the relative change of weight, BMI, TG, FM and BFP (All *P* < 0.05, Table 3). After adjusting for age and gender, serum uric acid was still negatively correlated with the relative change of weight, TG, FM and BFP (All *P* < 0.05, Table 3), but the correlation coefficient was decreased.

**Table 3:** The correlation between uric acid at baseline and relative changes of variables during weight loss

	Relative changes* (Δ, n = 52)	Uric acid (mmol/L)		Adjustment for Age and Sex	
		*r*	*P*	*r*	*P*
Height (cm)	3.12 ± 0.85	− 0.171	0.224	− 0.015	0.916
Weight (kg)	− 10.30 ± 2.83	− 0.448	0.001	− 0.358	0.011
BMI (kg/m^2^)	− 4.79 ± 0.89	− 0.304	0.028	− 0.260	0.068
WC(cm)	− 10.92 ± 4.35	− 0.158	0.264	− 0.083	0.565
HC (cm)	− 8.76 ± 3.03	− 0.265	0.058	− 0.241	0.092
WHR	− 0.02 (− 0.06, 0.00)	0.075	0.599	0.126	0.382
WHtR	− 0.08 ± 0.02	− 0.085	0.548	− 0.047	0.747
FBG (mmol/L)	− 0.38 (− 0.68, − 0.13)	0.012	0.931	0.062	0.668
FINS (pmol/L)	− 12.11 ± 6.56	− 0.135	0.340	− 0.088	0.543
HOMA-IR	− 2.89 ± 1.68	− 0.113	0.426	− 0.049	0.737
HOMA-β (%)	− 122.44 (− 205.41, − 84.04)	− 0.210	0.134	− 0.229	0.110
TC (mmol/L)	− 0.96 ± 0.73	− 0.231	0.100	− 0.158	0.275
TG (mmol/L)	− 0.85 ± 0.65	− 0.394	0.004	− 0.351	0.012
HDL (mmo/L)	− 0.09 (− 0.21, 0.00)	0.062	0.665	0.141	0.329
LDL (mmol/L)	− 0.52 (− 0.95, − 0.07)	− 0.055	0.697	− 0.050	0.730
UA (mmol/L)	− 53.50 (− 98.75, − 11.00)	− 0.578	< 0.001	− 0.067	< 0.001
FM (kg)	− 9.92 ± 2.78	− 0.545	< 0.001	− 0.474	0.001
FFM (kg)	0.28 (− 0.58, 1.39)	0.170	0.228	0.189	0.190
BFP (%)	− 7.23 ± 1.97	− 0.390	0.004	− 0.316	0.025
SBP (mmHg)	− 6.00 (− 14.75, 2.00)	− 0.167	0.237	− 0.115	0.426
DBP (mmHg)	− 7.50 (− 14.75, 3.00)	− 0.217	0.122	− 0.156	0.280

^*^The relative changes (Δ) = the value after intervention − the value before intervention; A positive value indicates a increase, and a negative value indicated an decrease

### General data and factors at baseline associated with fat mass loss

ΔFM ≥ 10 kg group (13.83 ± 1.58 years) was older than ΔFM < 10 kg group (12.72 ± 2.18 years), and the serum uric acid level (532.91 ± 98.50 vs 424.66 ± 88.53 mmol/L) was also higher than that of ΔFM < 10 kg group (both *P* < 0.05, Table 4). Compared with ΔFM < 10 kg group, ΔFM ≥ 10 kg group had significantly higher height, weight, BMI, WC, HC, WHR, WHtR, FM, FFM and BFR before intervention, and had worse glucose metabolism (all *P* < 0.05, Table 4). Furthermore, there was significant difference in FINS, TG, UA, SBP and DBP before intervention between the two FM groups (all *P* < 0.05, Table 4). However, multiple linear regression analysis showed that only UA before intervention were not conducive to the reduction of FM during weight loss (*P* < 0.05, Table 5).

**Table 4:** Comparison of variables at baseline with relative changes of fat mass

Baseline variables	ΔFM < 10 kg group (n = 29)	ΔFM ≥ 10 kg group (n = 23)	*P*
Age (year)	12.72 ± 2.18	13.83 ± 1.58	0.048
sex (male/ female)	15/14	19/4	0.038
Height (cm)	159.95 ± 7.27	170.10 ± 7.86	< 0.001
Weight (kg)	77.19 ± 16.27	99.63 ± 15.92	< 0.001
BMI (kg/m^2^)	29.95 ± 4.43	34.27 ± 3.59	< 0.001
WC(cm)	94.55 ± 11.89	106.45 ± 9.12	< 0.001
HC (cm)	102.83 ± 9.97	111.32 ± 8.00	0.002
WHR	0.92 ± 0.07	0.96 ± 0.05	0.043
WHtR	0.59 ± 0.06	0.63 ± 0.04	0.034
FBG (mmol/L)	4.95 ± 0.40	5.07 ± 0.50	0.341
FINS (pmol/L)	117.40 ± 48.05	162.26 ± 50.74	0.002
HOMA-IR	3.75 ± 1.64	5.29 ± 1.89	0.003
HOMA-β (%)	240.52 ± 109.35	320.39 ± 120.89	0.016
TC (mmol/L)	4.41 ± 0.88	4.56 ± 0.83	0.548
TG (mmol/L)	1.41 ± 0.53	1.86 ± 0.85	0.025
HDL (mmo/L)	1.22 ± 0.21	1.11 ± 0.24	0.099
LDL (mmol/L)	2.76 ± 0.63	2.95 ± 0.74	0.322
UA (mmol/L)	424.66 ± 88.53	532.91 ± 98.50	< 0.001
FM (kg)	32.73 ± 9.26	43.56 ± 9.93	< 0.001
FFM (kg)	44.45 ± 8.77	56.06 ± 9.53	< 0.001
BFP (%)	42.09 ± 5.01	43.60 ± 5.82	0.320
SBP (mmHg)	115.86 ± 10.92	126.83 ± 10.08	0.001
DBP (mmHg)	67.34 ± 10.09	72.96 ± 6.47	0.025

**Table 5:** Multiple linear regression of variables at baseline with relative changes of fat mass

Variables	Beta	t	*P*	95% CI
Age	0.104	0.751	0.458	− 0.247 to 0.538
Sex	0.026	0.190	0.850	− 1.431 to 1.727
Height	1.047	0.727	0.472	− 0.566 to 1.197
BMI	0.162	0.143	0.887	− 1.300 to 1.497
FM	0.395	0.397	0.694	− 0.415 to 0.617
FFM	0.601	0.615	0.543	− 0.359 to 0.670
SBP	0.047	0.460	0.648	− 0.038 to 0.061
DBP	0.001	0.004	0.996	− 0.071 to 0.072
WC	− 2.338	− 0.644	0.524	− 2.214 to 1.147
HC	− 0.509	− 0.284	0.778	− 1.152 to 0.869
WHR	0.065	0.051	0.959	− 105.009 to 110.461
WHtR	1.819	0.563	0.577	− 222.498 to 393.406
TG	− 0.097	− 0.792	0.433	− 1.328 to 0.582
FINS	0.165	0.924	0.362	− 0.010 to 0.027
HOMA-β	0.122	0.963	0.342	− 0.003 to 0.009
UA	0.296	2.778	0.009	0.002 to 0.013

## Discussion

In this study, we investigated the correlation between serum uric acid and body fat before and after weight loss in obese children and adolescents. The results showed that a combination of aerobic exercise and appropriate caloric control can help reduce weight, especially FM. Importantly, we found that UA was associated with BMI, FM, and FFM with adjusting age and gender, and played an important role in weight loss.

To date, the research on weight loss methods is roughly divided into dieting, exercise, meal replacement, drugs and surgical weight loss. Among them, exercise combined with diet intervention is considered to be the most scientific and reasonable way to lose weight [14, 15]. In terms of exercise methods, aerobic exercise is characterized by activities that are fun and have low exercise load intensity, which is more suitable for children. In addition, when the aerobic exercise time exceeds 30 min, the fat decomposition efficiency in the body is increased, and the body functions as a main energy supply material, thereby achieving the effect of weight loss [16]. Aerobic exercise not only helps to reduce fat and retain muscle [12], but also improves glucose and lipid metabolism [17, 18]. Likewise, our results showed that after a 6-weeks weight loss interventions, participants' weight and body fat were significantly reduced, while muscles were little increased. The average weight loss in obese children was 10.30 kg (12.07%) consisting of 9.92 kg of FM, which was more evident than in many other studies.

Globally, the incidence of hyperuricemia is estimated to be about 2.0–3.1%, and it is higher in men than in women [19–22]. With the increase in the incidence of childhood obesity, it is one of the reasons for the increase in the incidence of hyperuricemia in obese children [23]. Importantly, a 21-year follow-up study found that obese children were 3.25 times (male) and 3.55 times (female) more likely to develop hyperuricemia than adults of normal weight [3]. However, since the normal range of uric acid depends on age and gender [11, 12], the cut-off points for the diagnosis of hyperuricemia in children and adolescents are also different, which also brings some trouble for pediatricians to diagnose hyperuricemia.

Previous studies have shown that an elevated uric acid levels are associated with obesity, metabolic syndrome, hypertension and disorders of glucose and lipid metabolism in childhood [24, 25]. In addition, studies in adults indicated that hyperuricemia is positively associated with BMI, WC, and body fat, but is also positively associated with muscle [9, 26]. Surprisingly, the results from our study found that uric acid in obese children and adolescents was not only positively related to BMI, FM, and FFM, but also to the decrease in FM during weight loss. To the best of our knowledge, this result is first reported herein and is a highlight of our research.

However, the pathophysiological mechanism involved in the occurrence of hyperuricemia in obese children is not yet clearly established, and the mechanism of the effect of UA on body fat is also not yet fully understood. Previous study indicated that hyperuricemia induced inflammation of fat cells and oxidative stress. And fat cell inflammation and oxidative stress were the key mechanisms for fat mice to develop obesity and metabolic syndrome [27]. In addition, as for the association between blood uric acid and TC and TG, significant relationship was found in this study with adjusting for age and gender before and after intervention. This might imply that high uric acid affected the redistribution of adipose tissue by inducing the abnormality of lipids metabolism.

In light of the findings of this study, we should pay close attention to blood uric acid levels during weight loss in obese children, which may be a sensitive and useful signal indicator. However, for obese children diagnosed with hyperuricemia, whether uric acid lowering treatment is needed before weight loss should be considered and further researched. But it was worth mentioning that our research found that reasonable diet and exercise can reduce uric acid by 53 mmol/L.

Of course, the limitations of this study should be considered. First, the sample size of this study is small, and it is difficult to elaborate the mechanism of uric acid on body fat. Second, clinical data were only monitored and evaluated before and after the intervention. Finally, this study did not follow up on the effects of aerobic exercise combined with dietary weight loss on children's long-term quality of life and health status.

## Conclusions

In conclusion, a reasonable and professional aerobic exercise combined with proper diet control is of great significance for the reduction of body fat and muscle retention in obese children. It is found that a positive correlation between uric acid and body fat, but the higher the serum uric acid level before intervention, the more beneficial it is to reduce body fat. For this unexplainable results, it is worth studying what the reasonable range of serum uric acid is beneficial for the reduction of body fat at different age and gender. Therefore, in the future research, it is necessary to further explore the exact mechanism of the effect of uric acid on body fat through basic and clinical trials on the premise of expanding the sample size.

## Data Availability

All our materials and data are available. Data and materials are kept in the Department of Clinical Nutrition, Xinhua Hospital, Shanghai Jiaotong University School of Medicine.

## Abbreviations

BMI: Body mass index
FM: Fat mass
FFM: Free fat mass
BFP: Body fat percentage
WC: Waist circumference
HC: Hip circumference
WHR: Waist-to-hip ratio
WHtR: Waist-to-height ratio
SBP: Systemic blood pressure
DBP: Diastolic blood pressure
UA: Uric acid
FBG: Fasting blood glucose
FINS: Fasting insulin
TG: Triglyceride
TC: Total cholesterol
HDL-C: High density lipoprotein-cholesterol
LDL-C: Low density lipoprotein-cholesterol
HOMA‐IR: Homeostasis model assessment‐insulin resistance
HOMA‐β: Homeostasis model assessment‐β
